# Autophagy Induced by Micheliolide Alleviates Acute Irradiation-Induced Intestinal Injury *via* Inhibition of the NLRP3 Inflammasome

**DOI:** 10.3389/fphar.2021.773150

**Published:** 2022-01-18

**Authors:** Dong-ming Wu, Jing Li, Rong Shen, Jin Li, Ye Yu, Li Li, Shi-hua Deng, Teng Liu, Ting Zhang, Ying Xu, De-gui Wang

**Affiliations:** ^1^ School of Basic Medical Sciences, Lanzhou University, Lanzhou, China; ^2^ The First Affiliated Hospital of Chengdu Medical College, Chengdu, China; ^3^ School of Clinical Medicine, Chengdu Medical College, Chengdu, China

**Keywords:** autophagy, irradiation-induced intestinal injury, NLR pyrin domain 3, micheliolide, pyroptosis

## Abstract

Radiation-induced enteropathy (RIE) is one of the most common and fatal complications of abdominal radiotherapy, with no effective interventions available. Pyroptosis, a form of proinflammatory regulated cell death, was recently found to play a vital role in radiation-induced inflammation and may represent a novel therapeutic target for RIE. To investigate this, we found that micheliolide (MCL) exerted anti-radiation effects *in vitro*. Therefore, we investigated both the therapeutic effects of MCL in RIE and the possible mechanisms by which it may be therapeutic. We developed a mouse model of RIE by exposing C57BL/6J mice to abdominal irradiation. MCL treatment significantly ameliorated radiation-induced intestinal tissue damage, inflammatory cell infiltration, and proinflammatory cytokine release. In agreement with these observations, the beneficial effects of MCL treatment in RIE were abolished in *Becn1* ^*+/−*^ mice. Furthermore, super-resolution microscopy revealed a close association between NLR pyrin domain three and lysosome-associated membrane protein/light chain 3-positive vesicles following MCL treatment, suggesting that MCL facilitates phagocytosis of the NLR pyrin domain three inflammasome. In summary, MCL-mediated induction of autophagy can ameliorate RIE by NLR pyrin domain three inflammasome degradation and identify MCL as a novel therapy for RIE.

## Introduction

Radiotherapy is an important treatment strategy for several malignancies including lung cancer, prostate cancer, and renal cell carcinoma. However, despite significant improvements in radiotherapy delivery methods, incidence of irradiation induced bowel disease is a huge challenge for clinical, exposure to radiation causes lesions, which can result in several complications such as hematopoietic and gastrointestinal dysfunction and death (Seong, 2009; Hazell et al., 2020; Miccio et al., 2020; Sipaviciute et al., 2020; Molitoris et al., 2021). The small intestine is a highly radiosensitive organ, and radiation-induced enteropathy (RIE) tends to emerge quickly after radiation exposure. To date, RIE remains the most common and severe complication of the treatment of abdominal malignancies (Nussbaum et al., 1993; Lu et al., 2019). The well-known symptoms of RIE, including gastrointestinal hemorrhage, endotoxemia, bacterial infection, anorexia, nausea, vomiting, diarrhea, and loss of electrolytes and fluid, limit the therapeutic potential of radiotherapy in patients with abdominal malignancies and prevent further use of fractionated radiotherapy (Shadad et al., 2013; Hauer-Jensen et al., 2014). Patients with RIE appear nausea and vomiting, abdominal pain and diarrhea, tenesmus and other clinical symptoms, significantly reduce the life quality of patients, even cause serious complications such as colon stenosis, intestinal fibrosis and aggravate patients, greatly restricts the implementation of radiotherapy for patients with malignant tumors (Takemura et al., 2018; Akahane et al., 2020). Currently, the pathogenesis of RIE remains unclear, and there are no effective clinical interventions. Therefore, it is increasingly necessary to develop novel radioprotective agents with fewer adverse effects to protect patients from RIE.

The nucleotide-binding domain, leucine-rich repeat-containing receptor contains the NLR pyrin domain 3 (NLRP3) inflammasome, which is a cytosolic sensor of pathogens and endogenous damage-associated molecular patterns (Jiang et al., 2020; Seoane et al., 2020). Upon activation, NLRP3 facilitates the assembly of the apoptosis-associated speck-like protein containing a caspase recruitment domain (ASC) and the cysteine protease caspase-1 (Wang and Hauenstein, 2020). The formation of this complex cleaves the precursor of caspase-1 (pro-casp1) to its active form, caspase-1 (casp1-p20), and induces the cleavage of gasdermin D (GSDMD). This cascade results in pore formation on the plasma membrane and mediates a process known as pyroptosis (He et al., 2015; Ives et al., 2015). Pyroptosis is an inflammatory programmed cell death event that is distinct from apoptosis (Tang et al., 2020; Yu et al., 2021). Cells that undergo pyroptosis swell, release cytosolic contents, and release damage-associated molecular pattern molecules such as adenosine triphosphate, DNA, and the proinflammatory cytokines interleukin (IL)-18 and IL-1β into the extracellular milieu, thus initiating an inflammatory cascade in the affected tissue (Shi et al., 2017). Accumulating evidence suggests that NLRP3 inflammasome-mediated pyroptosis participates in radiation-induced damage and thus could be a novel therapeutic target for the treatment of RIE (Wei et al., 2019).

Autophagy is an intracellular self-digestive process that can deliver damaged organelles or proteins from the cytoplasm to lysosomes for degradation in order to maintain cellular homeostasis in response to external stimuli (Eskelinen, 2019; Haq et al., 2019; Pohl and Dikic, 2019). Numerous environmental stimuli, such as starvation or organelle damage, can induce autophagy (Haq et al., 2019). There is a growing appreciation that autophagy negatively regulates the activation of the NLRP3 inflammasome, thereby inhibiting inflammatory responses and reducing inflammatory injury of tissues in a disease state (Mehto et al., 2019; Fu et al., 2020; Peng et al., 2021). For example, it has been demonstrated that autophagy induced by the Axl receptor tyrosine kinase alleviates acute liver injury *via* inhibition of the NLRP3 inflammasome in mice (Han et al., 2016). Furthermore, administration of hydrogen-rich saline alleviated hyperpathia and microglial activation *via* autophagy-mediated inhibition of the NLRP3 inflammasome in a rat model of neuropathic pain (Chen et al., 2019). Collectively, these reports suggest that the modulation of autophagy in inflammatory conditions may be a novel strategy against RIE.

Radiation protective drugs for the prevention or treatment of RIE can significantly improve the life quality of patients (Wu et al., 2018). 441 compounds in Pyroptosis Compound Library (Selleck) was used in order to screening radiation protective drugs. Of which, Micheliolide (MCL) presents the strongest protective effect. MCL, isolated from the Michelia compressa and Michelia champaca plants, is a natural guaianolide sesquiterpene lactone derivative of parthenolide, and has shown promising anti-inflammatory, immunomodulatory, and therapeutic efficacy against multiple forms of cancer (Qin et al., 2016; Jiang et al., 2017; Sun et al., 2017). A previous study showed that MCL blocks doxorubicin-induced cardiotoxicity by ameliorating inflammation and necrosis through the repression of the PI3K/Akt/NF-kB signaling pathway (Kalantary-Charvadeh et al., 2019). Moreover, it was recently reported that MCL induces upregulation of peroxisome proliferator-activated receptor-γ expression, thereby alleviating NF-κB-mediated inflammation and activating autophagy in liver steatosis (Zhong et al., 2018). Based on these promising findings and the critical role that inflammation plays in RIE progression, we postulated that MCL may also play a promising role in the treatment of RIE. In the present study, we investigated the therapeutic effects of MCL in RIE, and evaluated the possible mechanisms by which it may be therapeutic.

## Materials and Methods

### Reagents

Pyroptosis Compound Library (L7400) were purchased from Selleck (Houston, TX, United States). Primary antibodies against NLRP3 (ab4207), GSDMD N-terminus (GSDMD-N; ab215203), caspase-1 (ab179515), LAMP-1 (ab208943), ASC (ab127537), P62 (ab109012), and beclin 1 (ab207612) were obtained from Abcam (Cambridge, MA, United States). The anti-light chain 3 (LC3) primary antibody (FNab04716) was obtained from Abcam Fine Test (Beijing, China). The horseradish peroxidase-conjugated secondary antibody (SA00001-1) and anti-glyceraldehyde-3-phosphate dehydrogenase antibody (60004-1-1g) were purchased from Proteintech (Wuhan, China). The following immunoglobulinG (H + L) antibodies were purchased from Beyotime Biotechnology (Shanghai, China): Cy3-labeled goat anti-rabbit (A0516), Cy3-labeled goat anti-mouse (A0521), Alexa Fluor 488-labeled goat anti-rabbit (A0423), and Alexa Fluor 488-labeled goat anti-mouse (A0428).

### Cell Culture and Radiation Treatment

The human intestinal epithelial cell (HIEC) line was purchased from the Cell Bank of the Chinese Academy of Sciences (Shanghai, China) and cultured in RPMI-1640 medium (Hyclone, Hudson, NH, United States) containing 10% fetal bovine serum and 1% penicillin-streptomycin in an incubator at 37°C, containing 5% carbon dioxide (CO_2_). Cells were treated with the desired dose (0, 5, 10, or 20 Gy) of X-ray irradiation at a rate of 2 Gy/min. Cell viability and lactate dehydrogenase (LDH) release assays were performed after irradiation for 24 or 48 h. In some experiments, HIECs were pretreated with different concentrations (0, 2.5, 5, or 10 μM) of MCL (Houston, TX, United States; s9309) for 2 h, and exposed to 10 Gy radiation. After 48 h, cell viability, LDH release, flow cytometry assays, and propidium iodide (PI) staining assays were performed.

### Cell Viability Analysis of an FDA-Approved Compound Library

A high throughput pyroptosis drug library was purchased from Selleck Chemicals (Houston, TX, United States). Compounds were stored as 10 mM stock solutions in dimethyl sulfoxide at 4 °C until use. HIECs in the logarithmic growth phase were plated in a 96-well plate at a density of 5 × 103 cells per well and incubated overnight in a cell incubator at 37°C and 5% CO_2_. Cells were treated with 10 μM of a compound for 2 h and then exposed to 10 Gy radiation. After 48 h, cell viability was measured using the cell counting kit-8 (Shanghai, China). Candidate drugs were selected based on the average cell viability in the replicate wells.

### LDH Release Assay

HIECs in the logarithmic growth phase were inoculated in a 96-well plate at a density of 5 × 103 cells per well and incubated overnight in a cell incubator at 37°C and 5% CO_2_. After receiving the corresponding treatment, LDH release assay was checked according to the operation steps of the LDH Cytotoxicity Assay Kit (Shanghai, China) instructions.

### Flow Cytometry

HIEC viability was measured by flow cytometry using an Annexin V-FITC/PI apoptosis detection kit (KeyGEN, Jiangsu, China; KGA1015-1018), as previously described (Li et al., 2020).

### PI Staining

HIECs were pretreated with different concentrations (0, 2.5, 5, or 10 μM) of MCL for 2 h and exposed to 10 Gy radiation. After 48 h, a PI solution (Shanghai, China) was added to the medium and further incubated for 30 min at 37°C in the dark.

### Animals and Irradiation

Wild-type (WT), *Nlrp3* ^*−/−*^ *,* and *Becn1* ^*+/−*^ mice on a C57BL/6J background were purchased from Beijing Weishanglide Biotechnology Co., Ltd. All mice were housed under the following conditions: 12-h light/dark cycle (lights on: 7:00, lights off: 19:00), temperature of 22 ± 2°C, humidity of 50 ± 10%, and standard diet and water. The WT mice were divided into the following groups (n = 15 per group): control, irradiation (IR), MCL (50 mg/kg), and MCL (10, 20, and 50 mg/kg)+IR. All *Nlrp3* ^*−/−*^ and *Becn1* ^*+/−*^ mice received IR either with or without MCL (50 mg/kg; n = 15 per group). An X-RAD 160-225 instrument (Precision X ray Inc, Branford, CT, United States; filter: 2 mm, AI; 42 cm, 225 kv/s, 12.4 mA, and 2.0 Gy/min) was used for abdominal irradiation. Except for the control and MCL groups, all other groups were exposed to 10 Gy radiation.

### MCL Treatment

For drug-treated groups, MCL was injected intraperitoneally (10, 20, and 50 mg/kg) 2 h before IR, and again daily for 5 days following IR. Mice were weighed every other day, and survival was recorded for 14 days. On day 8, the mice were sacrificed, and their serum was collected. The mice were then carefully and quickly dissected on ice trays, and their small intestines were removed to be used for subsequent analyses.

### Enzyme-Linked Immunosorbent Assay of Inflammatory Cytokines

Cell culture supernatant and mouse serum were collected, and the levels of IL-1β, IL-18, TNF-α, TGF-β1 and IFN-γ were detected using Enzyme-linked Immunosorbent assay kits (Shanghai, China) according to the kit instructions.

### Caspase-1 Activity Assay

Caspase-1 activity in mouse intestinal tissue was detected using the caspase-1 activity assay kit (Shanghai, China).

### Western Blotting

Protein samples were resolved by sodium dodecyl sulfate polyacrylamide gel electrophoresis (SDS-PAGE) on 12% gels, transferred to nitrocellulose membranes, blocked for 1 h at room temperature using Tris-buffered saline containing 0.1% Tween 20 and 5% fat-free milk, and probed with primary antibodies for 18 h at 4°C. Membranes were then stained at 37°C for 1 h with secondary antibodies conjugated with horseradish peroxidase, and immunoreactive signals were detected by enhanced chemiluminescence (SuperSignal; Pierce, Rockford, IL, United States). Protein signals were detected using the Chemi Doc XRS instrument plus Image Lab Software.

### Co-Immunoprecipitation Assays

The intestinal tissue lysates were incubated with primary antibody and shaken slowly overnight at 4°C. The next day, protein A + G agarose was added and shaken slowly at 4°C for 3 h. The samples were centrifuged, and the supernatant was aspirated. The pellet was washed five times with phosphate-buffered saline (PBS) containing 1× phenylmethyl-sulfonyl fluoride protease inhibitors. The supernatant was aspirated, and the pellet was resuspended in 1×SDS-PAGE electrophoresis loading buffer and incubated in a boiling water bath for 5 min. Samples were then used for SDS-PAGE electrophoresis.

### Hematoxylin and Eosin (H&E) Staining and Immunohistochemistry (IHC)

The small intestine tissue was fixed in 4% paraformaldehyde and embedded in paraffin after dehydration. Sections 4–6 μm thick were used for H&E staining or IHC. The sections were stained according to the instructions for the H&E staining kit (Beijing, China).

IHC was performed using an SPlink Detection Kit (ZSGB-BIO Technology Co., Ltd, Beijing, China). In short, the sections were deparaffinized and rehydrated, followed by antigen retrieval. Hydrogen peroxide was added to block the activity of endogenous peroxidase, and 5% goat serum was added dropwise for blocking. Sections were incubated in the primary antibody overnight at 4°C. The sections were the incubated at room temperature in peroxidase-labeled universal secondary antibody. Sections were then developed and counter-stained, and five high-powered field of view (10 × 40) images were randomly taken for each sample.

### Immunofluorescence (IF)

Sections were prepared as described above, and were then deparaffinized and rehydrated. Sections were blocked with 5% goat serum for 30 min at room temperature; thereafter, they were incubated overnight with the NLRP3 (1:100) and ASC (1:100) primary antibodies at 4°C. The next day, after washing with PBS, the sections were incubated in fluorescence-labeled secondary antibody solution for 2 h at 37°C, and washed again in PBS before staining with 4′,6-dimidyl-2-phenylindole for 5 min. Finally, slices were washed with PBS three times (5 min each time) and treated with anti-fluorescence quenching and sealing tablets. An Olympus inverted fluorescence microscope was used to capture fluorescence images over time.

### Confocal Microscopy

HIECs were seeded on sterile coverslips in a 24-well plate. MCL (50 pg/ml) was added to the wells for 2 h, followed by 10 Gy radiation treatment. IF staining was performed 48 h later, followed by 4’,6-dimidyl-2-phenylindole counterstaining. A confocal laser scanning microscope was used to collect the images.

### Cell Transfections

For NLRP3 silencing, HIEC cells were transfected with NLRP3 small interfering RNA (5′-AGA​AAT​GGA​TTG​AAG​TGA​AA-3′; RIBOBIO, Guangzhou, China), following the manufacturer’s instructions. The Opti-MEM (Gibco, Grand Island, NY, United States) transfection medium was replaced with a complete culture medium 5 h after transfection. All experiments were performed 48 h after transfection. The expression of NLRP3 was measured by real-time quantitative polymerase chain reaction (PCR).

### Real Time Fluorescent Quantitative PCR

Total RNA was extracted using an RNAprep FastPure kit (TSP413, TSINGKE, Shanghai, China), according to the manufacturer’s instructions. Total RNA was then reverse-transcribed using an RT6 cDNA synthesis kit (TSK302M, TSINGKE, Shanghai, China) to synthesize complementary DNA. Real-time fluorescent quantitative PCR was performed using a CFX96 Real-time System (Bio-Rad) with SYBR Green I (TSE202, TSINGKE, Shanghai, China). The 2-ΔΔCT method was used to calculate relative expression levels. The primer sense and antisense sequences were as follows: β-actin: 5′-CCTGGCACCCAGCACAAT-3′(sense); 5′-GGG​CCG​GAC​TCG​TCA​TAC-3′ (anti-sense). β-actin was used as an internal control for quantification. For NLRP3: F, 5′-GAGCTGGACCTCAGTGACAATGC-3′(sense); R, 5′-ACC​AAT​GCG​AGA​TCC​TGA​CAA​CAC-3′ (antisense).

### Statistical Analysis

All experiments were repeated independently at least three times. All animals were randomly assigned to experimental groups. Survival was analyzed using a log-rank test. Statistical significance among groups was determined using one-way analysis of variance or paired t-tests. Statistical significance was set at *p* < 0.05. Statistical analyses were performed using GraphPad Prism 7 (GraphPad Software, Inc, La Jolla, CA, United States).

## Results

### MCL Protects Against Ionizing Radiation in HIECs

**FIGURE 1:**
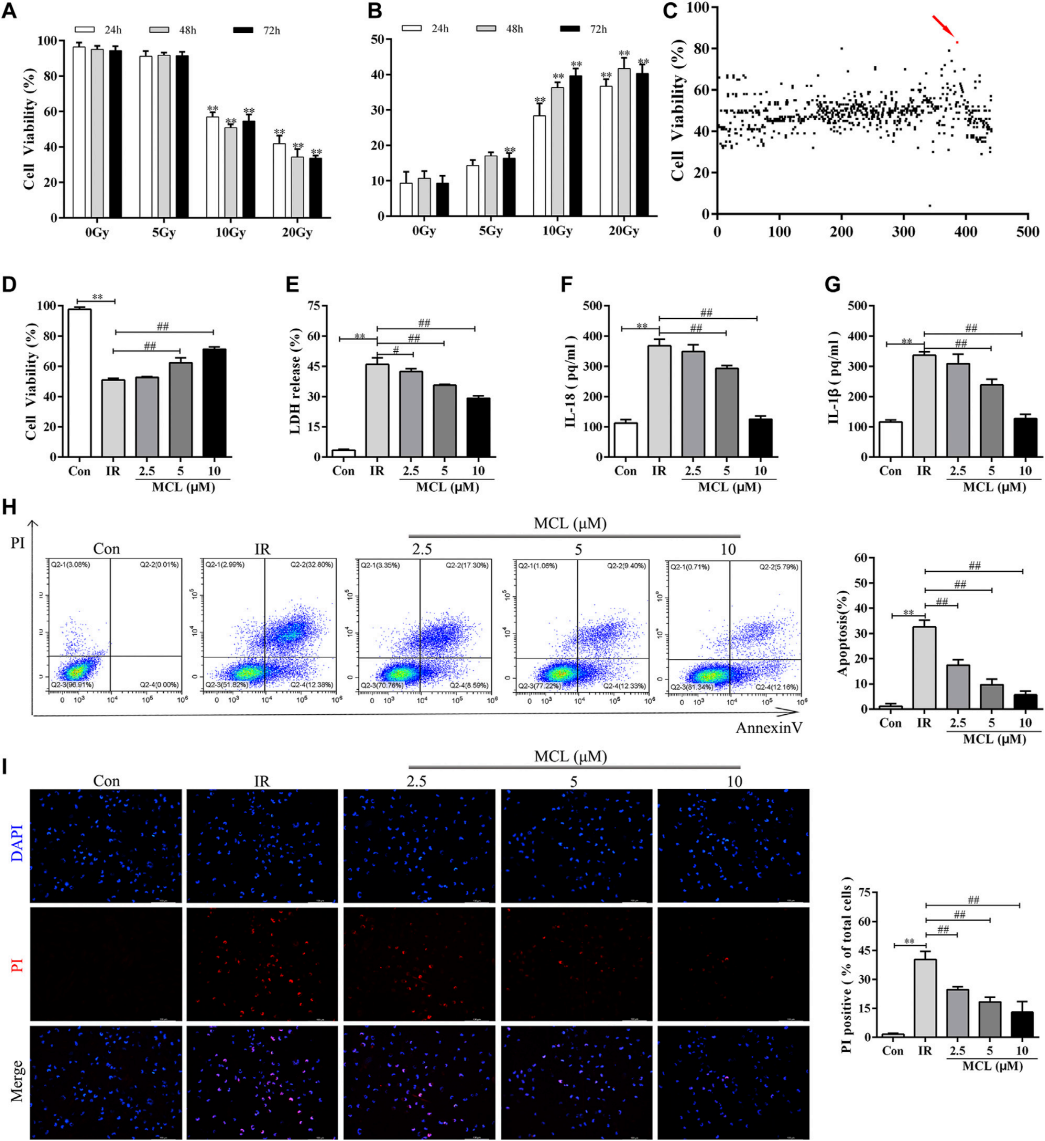
MCL protects against radiation in HIECs **(A)** Cell viability was significantly decreased in a dose-dependent manner following treatment with radiation (0, 5, 10, or 20 Gy) for 24, 48, or 72 h **(B)** LDH release increased in a time-and dose-dependent manner following radiation (0, 5, 10, or 20 Gy) **(C)** Cells were treated with 10 μM of the drug candidate library for 2 h and then exposed to radiation (10 Gy). Cell viability of HIECs exposed to the drug candidate library. Each point represents the percentage of cell viability of the compounds at a concentration of 10 μM **(D)** MCL increased cell viability following radiation (10 Gy) in a dose-dependent (0, 2.5, 5, or 10 μM) manner **(E)** MCL decreased LDH release following radiation (10 Gy) in a dose-dependent (0, 2.5, 5, or 10 μM) manner **(F)** MCL decreased cell culture supernatant IL-18 release following radiation (10 Gy) in a dose-dependent (0, 2.5, 5, or 10 μM) manner **(G)** MCL decreased cell culture supernatant IL-1β release following radiation (10 Gy) in a dose-dependent (0, 2.5, 5, or 10 μM) manner **(H)** HIECs were pretreated with different concentrations (0, 2.5, 5, or 10 μM) of MCL for 2 h and exposed to 10 Gy radiation. Representative flow cytometry scatter plots **(I)** HIECs were pretreated with different concentrations (0, 2.5, 5, or 10 μM) of MCL for 2 h and exposed to 10 Gy radiation. Representative propidium iodide staining fluorescence image. *p < 0.05, **p < 0.01, ***p < 0.001, ****p < 0.0001, two-tailed Student’s t-test. HIEC, human intestinal epithelial cell; LDH, lactate dehydrogenase; MCL, micheliolide; IL, interleukin.

To identify compounds with radioprotective effects, we first established a radiation-induced cell damage model in HIECs. We observed that cell viability was significantly decreased in a dose-dependent manner following treatment with radiation for 24, 48, or 72 h (Figure 1A, and lactate dehydrogenase (LDH) release increased in a time-and dose-dependent manner (Figure 1B). From these data, we chose a radiation dose of 10 Gy and a time point of 48 h for all subsequent experiments.

Next, we used the radiation-induced cell damage model to screen the compounds in the pyroptosis drug library. Among the 441 tested compounds, MCL elicited the strongest reversal of the reduced cell viability observed following radiation exposure (Figure 1C). Therefore, MCL was used in the subsequent experiments.

To further analyze the radioprotective effects of MCL, we pretreated HIECs with different concentrations of MCL prior to radiation exposure. Our results showed that cell viability with MCL exposure increased and LDH release decreased in a dose-dependent manner (Figure 1D,E). Furthermore, secretion of the inflammatory factors IL-18 and IL-1β decreased in a dose-dependent manner (Figure 1F,G). Moreover, the flow cytometry and PI staining indicate that 10 μM MCL is the most effective dose for promoting cell survival (Figure 1H,I). Altogether, these results indicate a protective role of MCL against radiation-induced cell damage.

### MCL Attenuates Radiation-Induced Intestinal Toxicity and Inflammatory Responses in Mice

**FIGURE 2:**
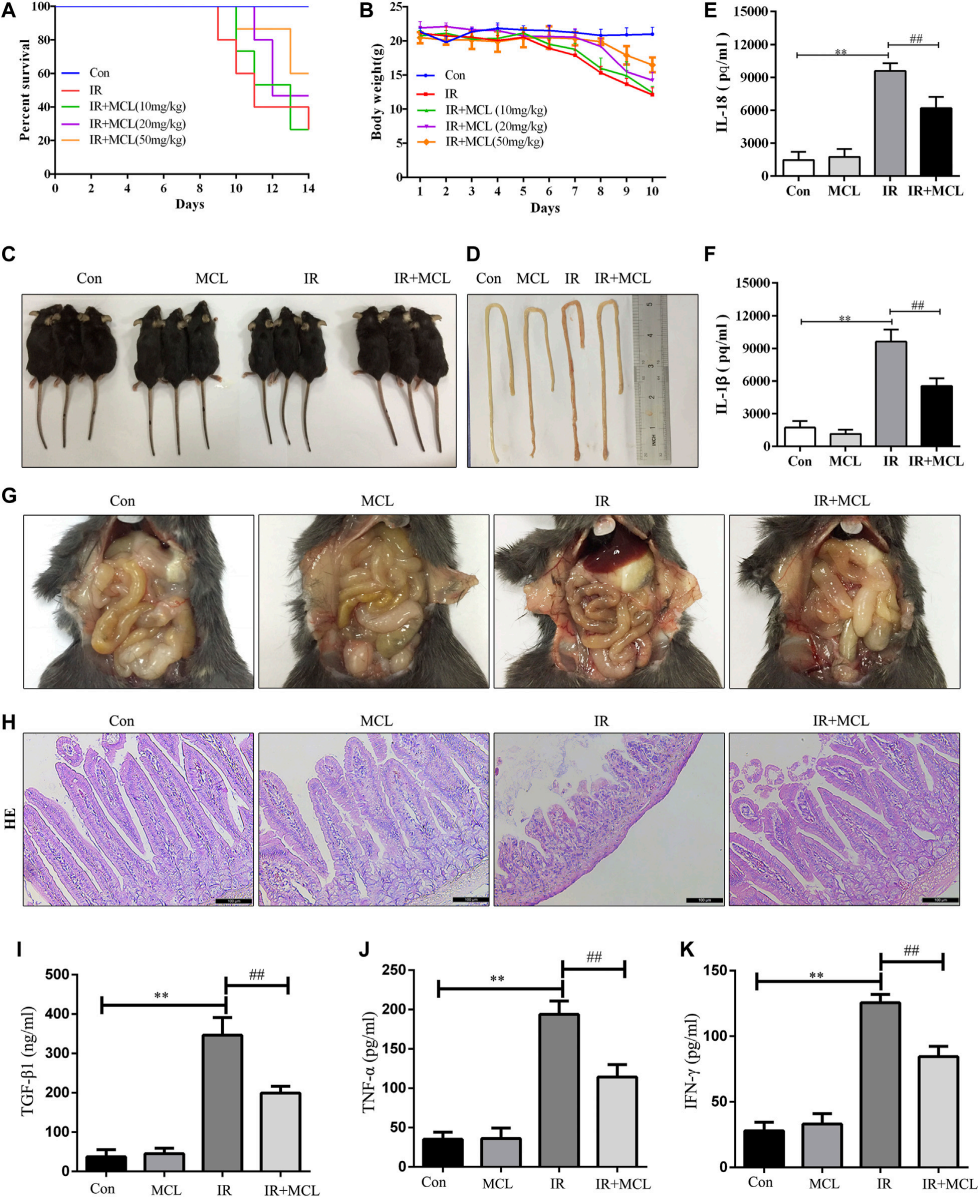
Micheliolide (MCL) attenuates radiation-induced intestinal toxicity and inflammatory responses in wild-type (WT) mice **(A)** Survival curves of all experimental groups following radiation **(B)** Weight loss following radiation exposure **(C)** Representative images of mice following radiation **(D, G)** Representative macroscopic appearance of the small intestine following radiation with MCL treatment **(E)** Serum interleukin (IL)-18 levels **(F)** Serum IL-1β levels **(I)** Serum TGF-β1 levels **(J)** Serum TNF-α levels **(K)** Serum IFN-γ levels **(H)** Representative intestinal hematoxylin and eosin staining. Con, untreated WT mice; IL, interleukin; IR, irradiation group; MCL (10 mg/kg)+ IR, mice pre-treated with MCL (10 mg/kg) prior to irradiation; MCL (20 mg/kg)+ IR, mice pre-treated with MCL (20 mg/kg) prior to irradiation; MCL (50 mg/kg)+ IR, mice pre-treated with MCL (50 mg/kg) prior to irradiation; MCL + IR, mice pre-treated with MCL (50 mg/kg) prior to irradiation; and MCL, mice treated with MCL (50 mg/kg) without irradiation. N = 15/group. *p < 0.05, **p < 0.01, ***p < 0.001, ****p < 0.0001, two-tailed Student’s t-test.

First, we tested the effect of MCL on the survival of WT C57BL/6 mice treated with a 10 Gy radiation dose. Mice in the IR group began to die by the ninth day after irradiation, with a survival rate of 26.6% on the 14th day (Figure 2A). The survival rates of MCL (10, 20, and 50 mg/kg) groups reached 26.7, 46.7, and 60%, respectively. Moreover, the survival rate of MCL 50 mg/kg group was significantly higher than that of other groups (*p* < 0.001; Figure 2A). Radiation-induced intestinal injury is characterized by diarrhea, sparse stools, and visible blood in the stool, which leads to significant weight loss. We observed that weight loss after radiation exposure was significantly attenuated in the MCL (50 mg/kg)+IR mice (Figure 2B,C). Therefore, an MCL dose of 50 mg/kg was used in subsequent experiments.

In the IR mice, histology, H&E staining revealed pathological changes including mucosal injury, necrosis, loss of tissue structure, edema, and inflammatory cell infiltration when compared with the control group (Figure 2D,H). MCL treatment significantly improved these pathological changes (Figure 2D,G,H). Furthermore, enzyme-linked immunosorbent assay analysis demonstrated that the secretion of IL-1β, IL-18,TGF-β1,TNF-αand IFN-γ in the blood was lower in the MCL (50 mg/kg)+IR group than the IR-group (Figure 2E,F,I–K). Altogether, these results suggest that MCL reduces the damage and inflammation caused by RIE. Importantly, MCL (50 mg/kg) administered alone without radiation had no significant effects on intestinal tissue, suggesting that there are no adverse effects of our experimental dose of MCL.

### MCL Inhibits Radiation-Induced NLRP3 Inflammasome-dependent Pyroptosis *in vitro*

**FIGURE 3:**
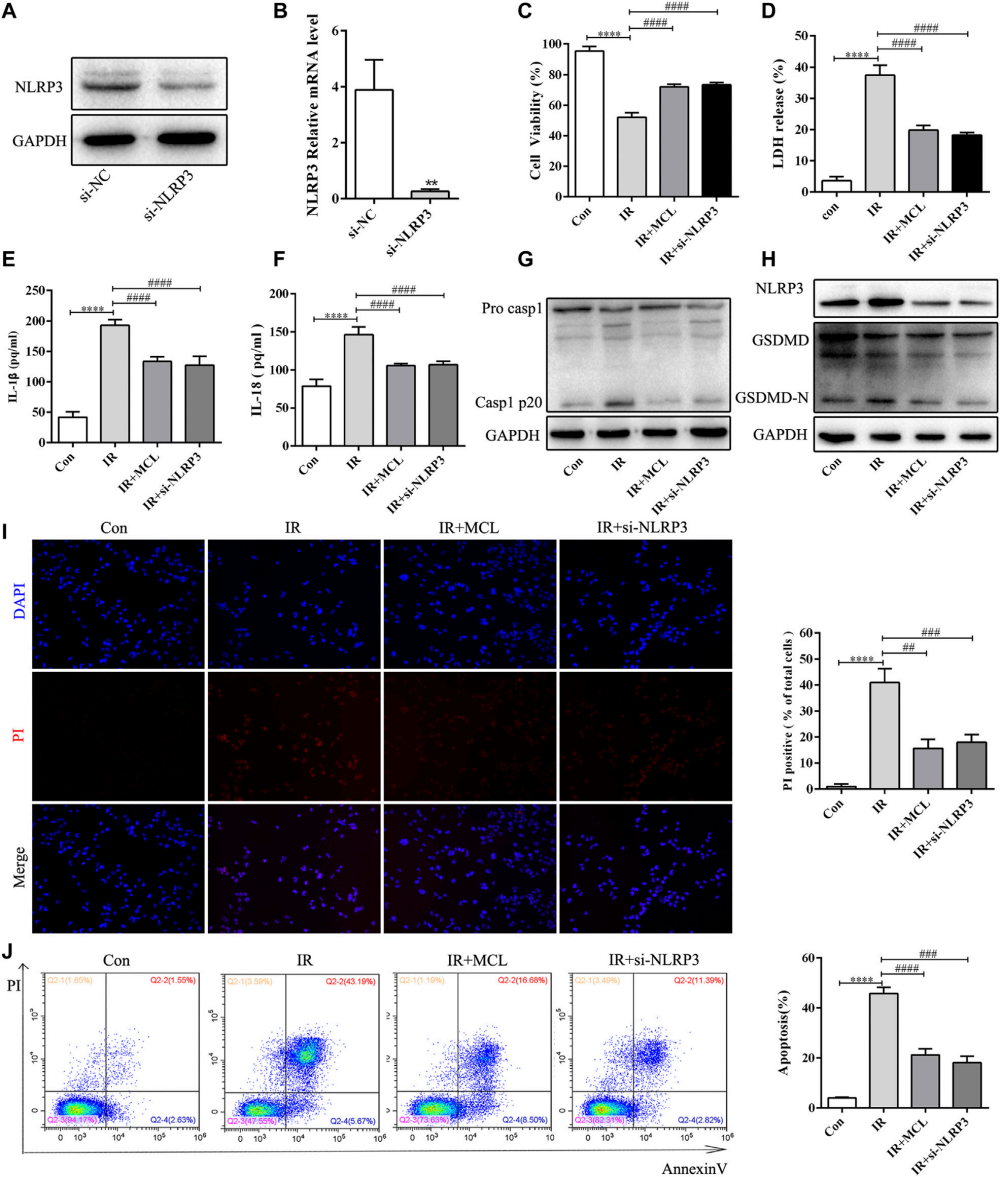
Micheliolide (MCL) inhibits radiation-induced NLRP3 inflammasome-dependent pyroptosis *in vitro* **(A,B)** Expression of NLRP3 in control (Ctl) and NLRP3-silenced (si-NLRP3) cells was detected by western blotting and quantitative real-time polymerase chain reaction **(C)** Cell viability was significantly increased by MCL (10 μM) treatment and NLRP3 knockout manner following treatment with radiation (10 Gy) for 48 h **(D)** LDH release was significantly decreased by MCL (10 μM) treatment and NLRP3 knockout manner following treatment with radiation (10 Gy) for 48 h **(E)** Cell culture supernatant IL-1β levels **(F)** Cell culture supernatant IL-18 levels **(G,H)** Expression of pro-casp1, casp1 p20, NLRP3, GSDMD, and GSDMD-N in si-NLRP3 cells and si-NC cells treated with MCL (10 μM) following irradiation (10 Gy) **(I)** Representative propidium iodide staining fluorescence image **(J)** Representative flow cytometry scatter plots. *p < 0.05, **p < 0.01, ***p < 0.001, ****p < 0.0001, two-tailed Student’s t-test. IL, interleukin; NLRP3, nucleotide binding domain leucine-rich repeat-containing receptor-pyrin domain containing three; GSDMD, gasdermin D.

To establish whether the activation of the NLRP3 inflammasome is involved in the underlying mechanism of RIE, we identified an effective RNAi oligonucleotide to silence NLRP3 expression in HIEC cells. We generated NLRP3-knockdown HIEC cell lines. NLRP3 knockdown was confirmed by western blot and real-time quantitative PCR (Figure 3A,B). We exposed HIEC/si-NLRP3 cells to 10 Gy of radiation and observed the cell viability after 48 h, and at the same time, detected the production of LDH, IL-1β, and IL-18 in the supernatant after 48 h. As expected, the HIEC/IR cell viability was significantly decreased (Figure 3C); the levels of LDH, IL-1β, and IL-18 in the HIEC/IR cells supernatant were dramatically increased compared with those in the control cells, whereas opposite effects were observed in HIEC/IR/si-NLRP3 cells and HIEC/IR/MCL (Figure 3D–F). In addition, GSDMD, which is essential for NLRP3-mediated pyroptosis, was cleaved by active caspase-1. In our study, we found that NLRP3 knockdown and MCL treatment suppressed the expression levels of active caspase-1 and GSDMD-N proteins (Figure 3H). PI staining also showed that IR exposure increased PI uptake by HIEC, and this increase was reduced by NLRP3 knockdown and MCL treatment (Figure 3I). Radiation-induced NLRP3 inflammasome-dependent pyroptosis was further confirmed by the flow cytometry results, which showed the inhibitory effect of MCL treatment on the proportion of double-positive HIEC (Figure 3J).

### MCL Inhibits Radiation-Induced NLRP3 Inflammasome-dependent Pyroptosis in Mice

**FIGURE 4:**
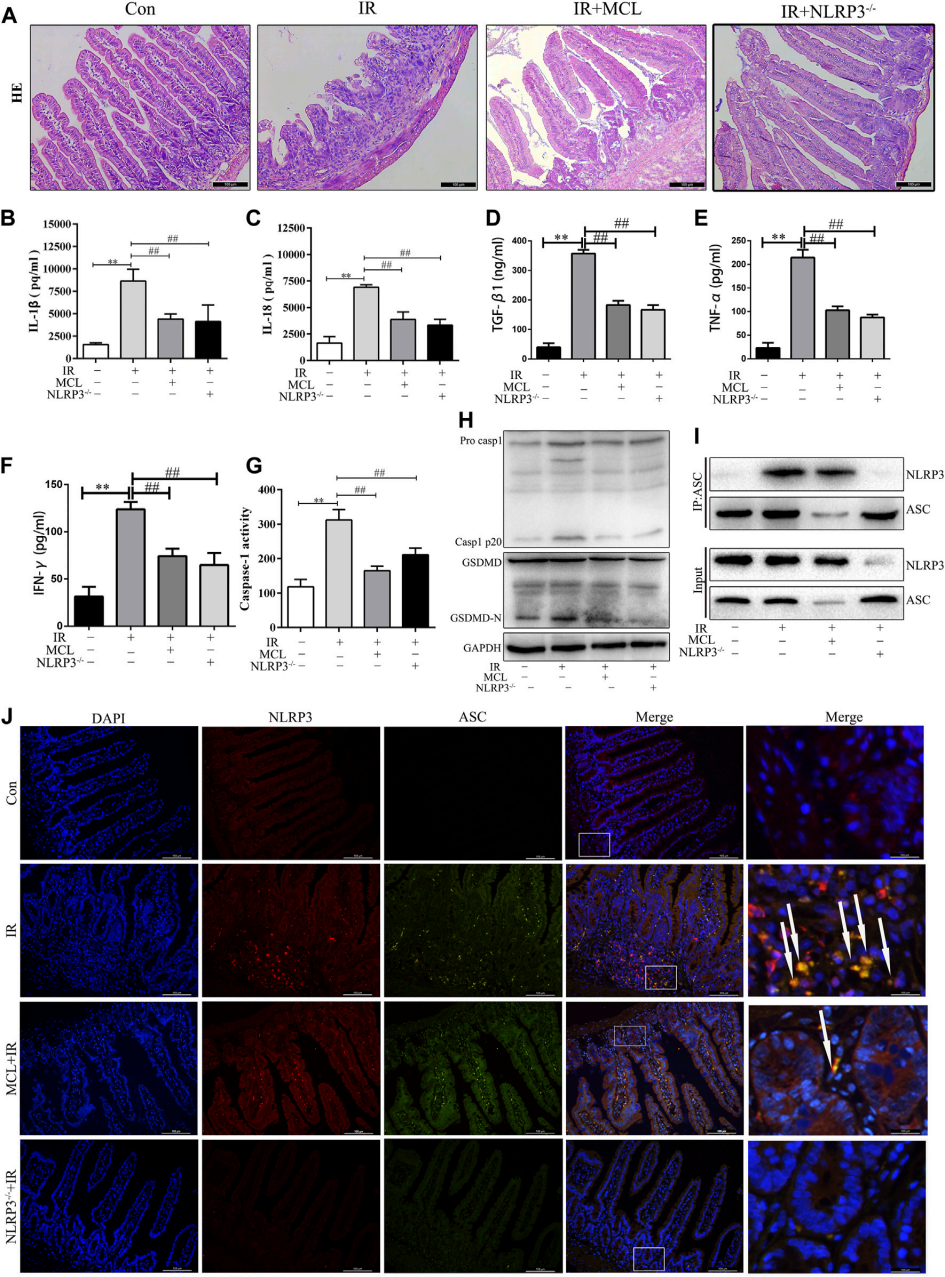
Micheliolide (MCL) inhibited irradiation-induced NLRP3 inflammasome activation in mice **(A)** Representative small intestine hematoxylin and eosin staining from NLRP3−/− and wild-type (WT) mice following irradiation **(B)** Serum IL-1β levels in NLRP3−/− and WT mice following irradiation **(C)** Serum IL-18 levels in NLRP3−/− and WT mice following irradiation (D) Serum TGF-β1 levels in NLRP3−/− and WT mice following irradiation **(E)** Serum TNF-α levels in NLRP3−/− and WT mice following irradiation (F) Serum IFN-γ levels in NLRP3−/− and WT mice following irradiation **(G)** Caspase-1 activity in the small intestine of NLRP3−/− and WT mice treated with MCL following irradiation (H) Expression of pro-casp1, casp1 p20, GSDMD, and GSDMD-N in NLRP3−/− and WT mice treated with MCL following irradiation **(I)** Formation of the NLRP3 and ASC complex in NLRP3−/− and WT mice treated with MCL following irradiation **(J)** Representative image of immunofluorescencent co-localization (arrows) between NLRP3 and ASC in small intestine tissue. *p < 0.05, **p < 0.01, ***p < 0.001, ****p < 0.0001, two-tailed Student’s t-test. Con, untreated WT mice; IR, WT mice that were treated with irradiation (10 Gy); MCL + IR, mice pre-treated with MCL prior to irradiation (10 Gy); NLRP3−/− + IR, NLRP3 knockout mice exposed to radiation (10 Gy); IL, interleukin; NLRP3, nucleotide binding domain leucine-rich repeat-containing receptor-pyrin domain containing three; GSDMD, gasdermin D. N = 15/group.

To establish whether the activation of the NLRP3 inflammasome is involved in the pathogenesis of RIE, NLRP3−/− and WT mice were administered abdominal radiotherapy at a dose of 10 Gy. NLRP3−/− mice exhibited significantly reduced inflammation following radiation than their WT counterparts (Figure 4A). Figure 4 show that after radiation of *NLRP3* knockout mice, the levels of IL-1β, IL-18, TGF-β1, TNF-α and IFN-γ (Figure 2B–F) in the blood were significantly lower in *NLRP3* ^−/−^ mice than in WT mice after radiation. These results indicate that the activation of the NLRP3 inflammasome contributes to the inflammation observed in RIE and may promote RIE progression after irradiation.

Next, we evaluated whether MCL exerted anti-inflammatory effects through inhibition of the NLRP3 inflammasome. As shown in Figure 4, the activity of caspase-1 (Figure 4G)and the expression of cleaved caspase-1 and GSDMD-N proteins (Figure 4H) were significantly increased in IR-treated WT mice. MCL-treated WT mice and *NLRP3* ^−/−^ mice exhibited reduced expression of these proteins after radiation. In addition, co-immunoprecipitation assays revealed that the NLRP3-ASC complex was increased significantly in the intestinal tissue of IR-treated WT animals (Figure 4I). This finding was confirmed with IF, in which the number of cells positive for both NLRP3 and ASC was increased in IR-treated animals (Figure 4J). MCL-treated WT animals and NLRP3−/− mice exhibited a significantly reduced assembly of the NLRP3 inflammasome following radiation (Figure 4I,J).

### MCL Enhances Autophagy in Mice Intestinal Tissue and HIECs After Radiotherapy

**FIGURE 5:**
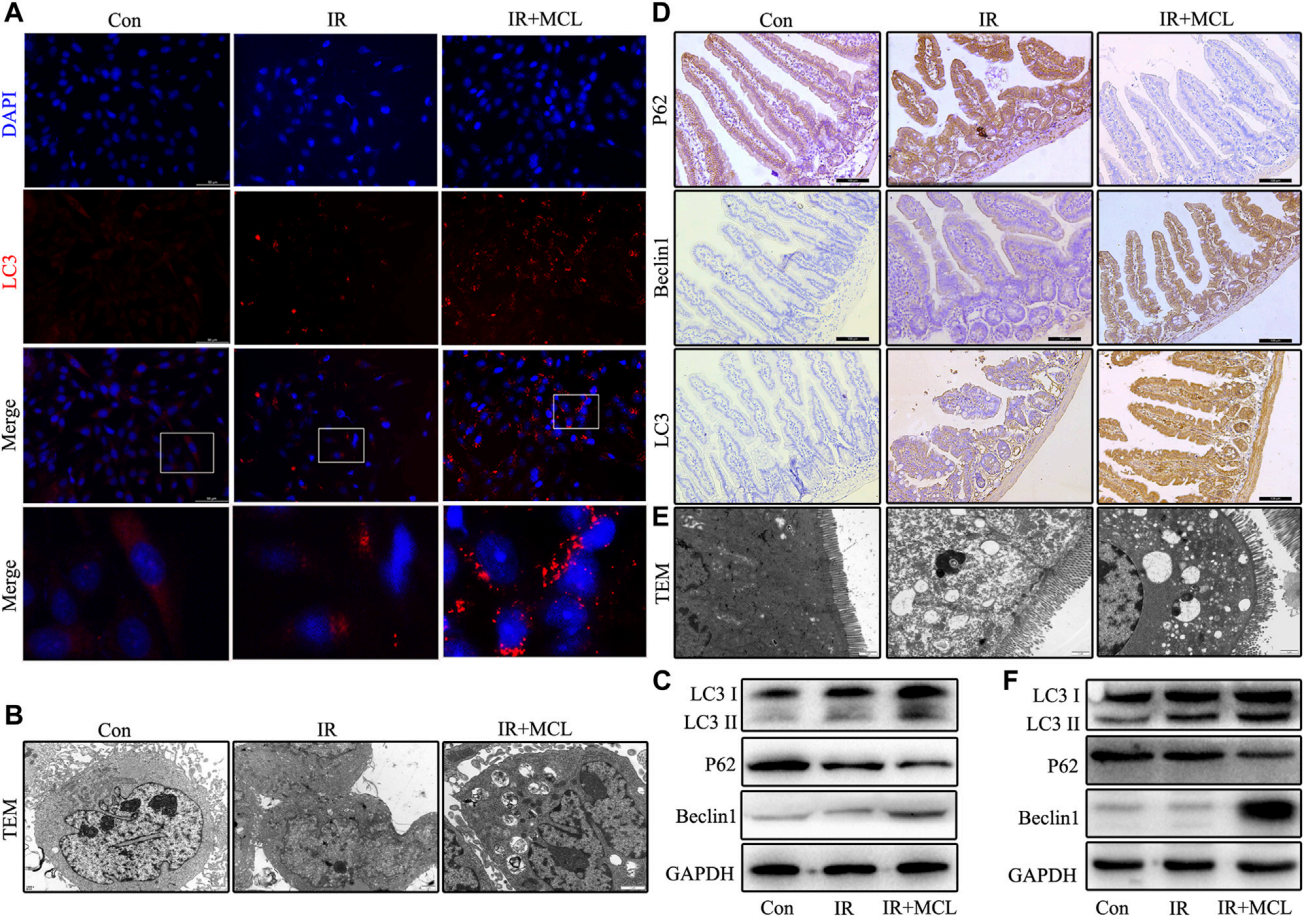
MCL enhances autophagy in mice intestinal tissue and HIEC after radiotherapy. Con, untreated HIECs; IR, HIECs that were treated with irradiation (10 Gy); MCL + IR, HIECs pre-treated with MCL (10 μM) prior to irradiation **(A)** Representative immunofluorescence image of LC3 aggregation in HIECs **(B)** Autophagosomes (arrows) in HIEC detected by transmission electron microscopy **(C)** Western blot of p62, beclin 1, and LC3 protein expression in HIEC. Con, untreated WT mice; IR, WT mice that were treated with irradiation (10 Gy); MCL + IR, WT mice pre-treated with MCL (50 mg/kg) prior to irradiation (10 Gy) **(D)** Immunohistochemical staining of p62, beclin 1, and LC3B in small intestine tissues **(E)** Autophagosomes (arrows) in small intestine tissue detected by transmission electron microscopy **(F)** Western blot of p62, beclin 1, and LC3 protein expression in small intestine tissues. *p < 0.05, **p < 0.01, ***p < 0.001, ****p < 0.0001, two-tailed Student’s t-test. IR, irradiation; HIEC, human intestinal epithelial cell; MCL, micheliolide.

To further explore whether MCL can regulate autophagy, we performed IF to detect the aggregation of the autophagosome component LC3. When compared with IR-only HIEC, the aggregation levels of LC3 significantly increased in the MCL + IR group (Figure 5A). Transmission electron microscopy was employed to detect autophagosomes and autolysosomes in the HIEC and the intestinal tissue. The number of autophagosomes was increased in the MCL + IR group compared with IR-only HIEC and mice (Figure 5B,E). In addition, we performed western blot to detect the expression levels of autophagy markers LC3, p62, and beclin 1 in the HIEC. The levels of LC3 and beclin 1 were higher, and levels of p62 were lower in the HIEC of MCL + IR-treated than in the HIEC of IR-treated, indicating that MCL treatment significantly increased autophagy (Figure 5C). Furthermore, IHC and western blot analysis of the same proteins recapitulated these results in mouse intestinal tissues (Figure 5D,F). Altogether, these results indicate that MCL enhances autophagy in mouse intestinal tissues following radiation.

### MCL Mediates Autophagy of the NLRP3 Inflammasome

**FIGURE 6:**
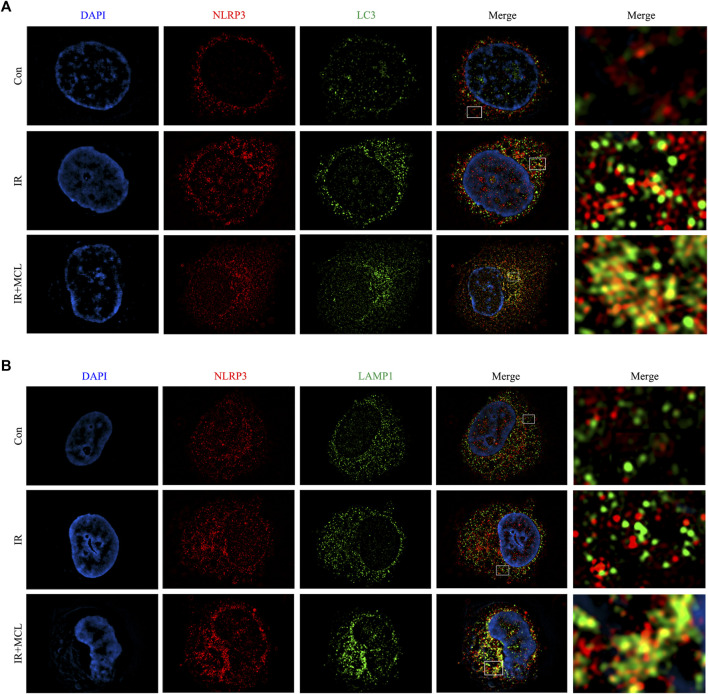
Micheliolide (MCL) mediates autophagy of the NLRP3 inflammasome **(A–B)** HIECs were stained for LC3/LAMP1 (green) and NLRP3 (red) and analyzed by confocal microscopy. Co-localization of NLRP3 (arrows) and LC3/LAMP1 (arrowheads) is apparent. *p < 0.05, **p < 0.01, ***p < 0.001, ****p < 0.0001, two-tailed Student’s t-test. Con, untreated HIECs; IR, HIECs exposed to radiation (10 Gy); IR + MCL, HIECs pretreated with MCL (10 μM) prior to radiation (10 Gy) exposure; HIEC, human intestinal epithelial cell; LC3/LAMP1, light chain three/lysosome-associated membrane protein.

After the autophagosome matures, lysosomes and autophagosomes fuse to form an autophagolysosome, which leads to the degradation of the autophagosome (Zhao and Zang, 2019). To determine whether autophagy is involved in the degradation of the NLRP3 inflammasome, we evaluated the co-localization of NLRP3 with LC3 and lysosome-associated membrane protein (LAMP-1). Fluorescent confocal microscopy showed that NLRP3 and LC3 were co-localized, and NLRP3 was surrounded by the lysosomal marker LAMP-1, suggesting that the NLRP3 inflammasome is fused with autophagosomes and lysosomes (Figure 6A,B). These data indicate that MCL can promote the formation of autophagic lysosomes which degrade NLRP3 inflammasomes.

### MCL Inhibits the NLRP3 Inflammasome via the Activation of Autophagy

**FIGURE 7:**
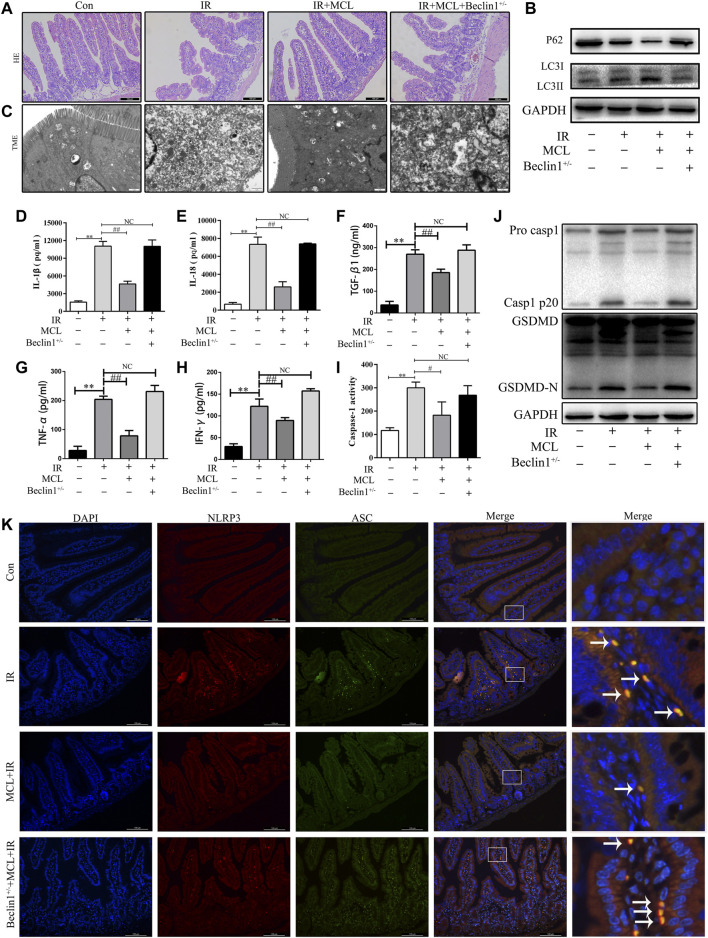
Micheliolide (MCL) inhibits the NLRP3 inflammasome via the activation of autophagy **(A)** Representative small intestine hematoxylin and eosin staining from Beclin1+/-and wild-type (WT) mice following irradiation **(B)** Western blot of p62 and LC3 protein expression **(C)** Autophagosomes in small intestine tissue detected by transmission electron microscopy **(D)** Serum IL-1β levels in MCL-treated heterozygous beclin 1 knockout mice following radiation **(E)** Serum IL-18 levels in MCL-treated heterozygous beclin 1 knockout mice following radiation **(F)** Serum TGF-β1 levels in MCL-treated heterozygous beclin 1 knockout mice following radiation **(G)** Serum TNF-α levels in MCL-treated heterozygous beclin 1 knockout mice following radiation **(H)** Serum IFN-γ levels in MCL-treated heterozygous beclin 1 knockout mice following radiation **(I)** Caspase-1 activation in the small intestine **(J)** Expression of pro casp1, casp1 p20, GSDMD, and GSDMD-N was examined by western blot in MCL-treated heterozygous beclin 1 knockout mice. **(K)** Representative immunofluorescence image of the co-localization (arrows) between NLRP3 and ASC in the small intestine tissue. *p < 0.05, **p < 0.01, ***p < 0.001, ****p < 0.0001, two-tailed Student’s t-test. Con, untreated WT mice; IR, WT mice that were treated with irradiation (10 Gy); MCL + IR, mice pre-treated with MCL (50 mg/kg) prior to irradiation (10 Gy); MCL + IR + Beclin1^+/−^, Beclin1+/-mice with MCL (50 mg/kg) prior to irradiation (10 Gy); IL, interleukin; NLRP3, nucleotide binding domain leucine-rich repeat-containing receptor-pyrin domain containing three; GSDMD, gasdermin D; LC3, anti-light chain 3.

To evaluate the role of autophagy in MCL-mediated protection following radiation, we examined whether MCL was protective against RIE in heterozygous beclin-1 knockout mice (*Becn* ^*+/*-^). We observed that the MCL-mediated protection against histopathological changes following radiation was abolished in *Becn* ^*+/-*^ mice (Figure 7A). Furthermore, MCL-induced autophagy following radiation was abolished in *Becn+/-*mice (Figure 7B,C). Moreover, heterozygous knockout of beclin 1 reversed the MCL-mediated downregulation of IL-1β, IL-18, TGF-β1, TNF-α and IFN-γ following radiation (Figure 7D–H).

To determine whether MCL-mediated suppression of the NLRP3 inflammasome is dependent on autophagy, we evaluated the expression of NLRP3-associated proteins in *Becn* ^*+/-*^ mice. Similar to previous experiments, the expression of GSDMD-N and casp1 p20 was increased following radiation in WT mice, an effect which was inhibited by MCL treatment (Figure 7J). In contrast, MCL-mediated inhibition of caspase 1 activity (Figure 7I), and expression of GSDMD-N and casp1 p20 was abolished in *Becn* ^*+/-*^ mice (Figure 7J). Concordantly, co-localization of NLRP3 with ASC was observed in MCL-treated *Becn* ^*+/-*^ mice following radiation (Figure 7K). Altogether, these data indicate that MCL inhibits the activation of the NLRP3 inflammasome in an autophagy-dependent manner.

## Discussion

Radiotherapy is regarded as a vital treatment for abdominal and pelvic tumors (Miccio et al., 2020). However, the intestines are relatively sensitive to irradiation, and normal tissues surrounding the tumor, especially the small intestines, may be damaged by radiation exposure (Lu et al., 2019). Currently, there are no effective therapeutic agents to prevent intestinal injury resulting from radiotherapy in cancer patients (Hauer-Jensen et al., 2014). Therefore, novel compounds that can prevent or reverse RIE are urgently needed. In this study, we screened a drug library and identified MCL as a potentially therapeutic agent for RIE. We demonstrated that MCL effectively inhibited NLRP3 inflammasome activity and blocked pyroptosis in RIE, which may be associated with the ability of MCL to induce autophagy of the NLRP3 inflammasome.

In a previous study, it was demonstrated that pyroptosis plays an important role in radiation-induced inflammatory injury (Wu et al., 2018). The intact intestinal epithelium acts as an effective barrier against external stimuli, regulates intestinal homeostasis, and is sensitive to radiotherapy (Kessler et al., 2018). Therefore, we screened the pyroptosis-drug compound library in a HIEC model of radiation-induced intestinal injury. Of all the drugs in this library, MCL was found to exert the strongest effect on cell viability following radiation exposure. Furthermore, our results showed that MCL dose-dependently inhibited radiation-induced cell death and LDH release. Based on these findings, we hypothesized that MCL would attenuate radiation-induced intestinal injury in an animal model. To test this hypothesis, we exposed WT C57BL/6J mice to 10 Gy of radiation and evaluated the subsequent inflammatory response and cell damage in the intestinal tissue. The data gathered indicated that irradiation induces an inflammatory reaction accompanied by abundant production of pro-inflammatory cytokines, including IL-1β and IL-18. Interestingly, MCL alleviated radiation-induced intestinal damage and downregulated cytokine levels, consistent with previous reports (Liu et al., 2019; Tian et al., 2020).

The NLRP3 inflammasome is a multi-protein platform. It has been shown that the NLRP3-associated protein, caspase-1, is activated upon cellular infection or stress and leads to a form of programmed cell death called pyroptosis (Luan et al., 2018; Wang & Hauenstein, 2020). In the process of pyroptosis, cleaved caspase-1 specifically cleaves the linker between GSDMD-N and the carboxy-terminal domain of GSDMD, triggering the secretion of IL-1β and IL-18, which are required for pyroptosis and expansion of the inflammatory reaction (Ives et al., 2015; Kovacs and Miao, 2017). A recent study reported that radiation exposure induces NLRP3 inflammasome pathway activation, which participates in the inflammatory response to radiation damage in mouse lungs and intestines (Wei et al., 2019). Based on these findings, we speculated that MCL would mitigate RIE in mice through the inhibition of the NLRP3 inflammasome and pyroptosis. We observed that the damaging responses to radiation were attenuated in NLRP3−/− mice, suggesting NLRP3 involvement in RIE-induced damage. Furthermore, MCL treatment reduced the expression of Casp1 p20 and GSDMD-N, thereby inhibiting pyroptosis in RIE, suggesting that the therapeutic effects of MCL may be mediated by inhibition of the NLRP3 inflammasome (Gong et al., 2019; Sui et al., 2020).

The autophagy pathway is known to play a crucial role in regulating inflammation (Cao et al., 2019). Impaired autophagy may contribute to the aberrant activation of signaling pathways, leading to uncontrolled inflammation and cell death (Han et al., 2019). An increasing number of studies have indicated that the modulation of autophagy could protect against multiple organ injuries by inhibiting the NLRP3 inflammasome (Chen et al., 2020; Qiu et al., 2020; Peng et al., 2021). Moreover, research suggests that MCL mitigates the severity of liver steatosis by enhancing autophagy and attenuating NF-κB-mediated inflammation (Zhong et al., 2018). Additionally, it has been reported that resveratrol can inhibit the activation of the NLRP3 inflammasome by inducing autophagy, which ameliorates IgA nephropathy (Wu et al., 2020; Chen et al., 2021). Considering this evidence, we speculated that MCL may alleviate radiation-induced pyroptosis by promoting autophagy and subsequently inhibiting the activation of the NLRP3 inflammasome. Therefore, we evaluated the involvement of several autophagy-related genes in this study. BECN1, encoding beclin 1, is a protein essential for autophagy, which acts in cooperation with the PtdIns3K pathway to enhance the formation of the autophagic vacuole. We found that the heterozygous deletion of beclin 1 in mice reduces the radioprotective capacity of MCL. Furthermore, the inhibition of NLRP3-ASC co-localization by MCL was absent in *Becn1* ^*+/−*^ mice. Collectively, these results suggest that MCL may regulate autophagy to inhibit the NLRP3 inflammasome and exert a radioprotective effect.

No research performed to date has evaluated the effect of MCL on the relationship between the NLRP3 inflammasome and autophagy. LC3 is a protein associated with phagophores, the precursor to the autophagosome (Kabeya et al., 2000; Wirawan et al., 2012; Katsuragi et al., 2015). Previous studies have described the clearance of the NLRP3 inflammasome via autophagy. In this process, the NLRP3 inflammasome is entirely enclosed by LC3 and delivered to lysosomes for destruction (Mehto et al., 2019; Biasizzo and Kopitar-Jerala, 2020). Given this evidence, we hypothesized that MCL would promote targeting of NLRP3 inflammasomes to autophagolysosomes, thereby increasing their degradation. Direct visualization by super-resolution microscopy in our HIEC model of RIE confirmed that NLRP3 is completely enclosed by LC3 positive structures, which was increased with MCL treatment. Furthermore, we demonstrated the direct co-localization of NLRP3 with the lysosomal protein LAMP1. Altogether, these results indicate that MCL attenuates RIE by targeting NLRP3 inflammasomes for degradation.

Nonetheless, this study has some limitations. We did not determine other potential molecular targets of MCL in this study, and we were unable to determine whether NLRP3 can bind to multitarget proteins involved in inflammatory or autophagy processes. Future studies should evaluate the specific target proteins of NLRP3 binding.

In summary, we demonstrated that MCL ameliorates RIE via inhibition of the NLRP3 inflammasome, which is likely a result of enhanced autophagy leading to NLRP3 inflammasome degradation. These data suggest that MCL may be a novel drug candidate for the treatment of RIE.

## Data Availability

The original contributions presented in the study are included in the article/supplementary material, further inquiries can be directed to the corresponding authors.
